# Ageing results in an exacerbated inflammatory response to LPS by resident lung cells

**DOI:** 10.1186/s12979-024-00467-8

**Published:** 2024-09-12

**Authors:** Celia Diaz-Nicieza, Laura Sahyoun, Christina Michalaki, Cecilia Johansson, Fiona J. Culley

**Affiliations:** National Heart and Lung Institute, Faculty of Medicine, Imperial College London, Norfolk Place, London, W2 1PG UK

**Keywords:** Ageing, Lung, Infection, Inflammation, Innate immunity

## Abstract

**Background:**

Ageing is associated with an increased risk of lung infection and chronic inflammatory lung disease. Innate immune responses are the first line of defence in the respiratory tract, however, age-related changes to innate immunity in the lung are not fully described. Both resident haematopoietic cells, such as alveolar macrophages, and non-haematopoeitic cells, such as epithelial and endothelial cells can contribute to inflammatory and immune responses in the lung. In this study we aimed to determine the impact of ageing on early innate responses of resident cells in the lung.

**Results:**

Aged and young mice were inoculated intranasally with lipopolysaccharide (LPS). After 4 h, aged mice recruited higher numbers of neutrophils to the airways and lung. This exacerbated inflammatory response was associated with higher concentrations of chemokines CXCL1, CXCL2 and CCL2 in the airways. Next, precision cut lung slices (PCLS) were stimulated ex vivo with LPS for 16 h. Gene expression of *Cxcl2*,* Tnf* and *Il1b* were all higher in PCLS from aged than young mice and higher levels of secretion of CXCL2 and TNF were detected. To determine which lung cells were altered by age, LPS was intranasally administered to aged and young mice and individual populations of cells isolated by FACS. RT-PCR on sorted cell populations demonstrated higher expression of inflammatory cytokines *Cxcl2*,* Ccl2* and *Tnf* in epithelial cells and alveolar macrophages and higher expression of *Cxcl2* by endothelial cells of aged mice compared to young. These differences in expression of pro-inflammatory cytokines did not correspond to higher levels of *Tlr4* expression.

**Conclusions:**

Ageing leads to a heightened neutrophilic inflammatory response in the lung after LPS exposure, and higher expression and production of pro-inflammatory cytokines by resident lung cells, including alveolar macrophages, epithelial cells and endothelial cells. The responses of multiple resident lung cell populations are altered by aging and contribute to the exacerbated inflammation in the lung following LPS challenge. This has implications for our understanding of respiratory infections and inflammation in older people.

**Supplementary Information:**

The online version contains supplementary material available at https://doi.org/10.1186/s12979-024-00467-8.

## Background

Older adults are at increased risk of respiratory infections, chronic lung conditions and lung cancer [1–3]. Innate immune responses in the lung are an important first line of defence against infection and are central to regulation of respiratory immunity and resolution of inflammation. Ineffective or excessive lung inflammation can lead to susceptibility to infection and airway damage and occlusion. However, the changes to innate respiratory immunity with age are relatively poorly understood [3].

Cells that contribute to the lung innate immune response include resident haematopoeitic and non-haematopoeitic cells and these populations undergo changes in both function and composition in the aged lung [3–6]. Alveolar macrophages (AMs) are an abundant resident cell population found in the lower airways where they interact closely with the epithelium to regulate inflammation [7]. In infection, they play key roles in initiation and resolution of inflammation. Numbers of AMs reduce with age and while some studies have reported an age-related decline in functions of AMs, including phagocytosis and cytokine production, others report a more pro-inflammatory phenotype [8–14].

Structural, non-haematopoeitic cells, including epithelial, endothelial and stromal cells, such as fibroblasts, also contribute to innate immunity in the lung [5], but the age-related changes in these populations are less well described. In addition to forming a physical barrier to infection, the respiratory epithelium regulates the function of AMs and responds to the presence of foreign pathogens by secretion of cytokines and chemokines, which in turn activate local immune cells and recruit circulating leukocytes [15]. With age, mucociliary clearance is impaired and numbers of epithelial progenitor cells decline [3, 16, 17]. Distal lung type II alveolar epithelial cells (AEC2) exhibit age-related changes in expression of immune genes, including those related to MHC class I, and increased production of pro-inflammatory mediators during infection [4, 6, 18, 19]. The contribution of other structural cells, such as the endothelium and other stromal cells, to immune responses in the lung is increasingly recognized [5, 20, 21]. Age-related changes to endothelial regeneration have been associated with impaired resolution of inflammation in the lung [22]. However, how the response to immune stimuli of these structural cells changes with age has not been fully described.

There is an important need to fully understand how the differences in cell populations contribute to inflammation in the lung with age. Studies of isolated cells ex vivo may not lead to a full understanding of age-related changes to innate immune response in the lung, as multiple complex interactions between resident cells and the lung environment will determine the outcome of infectious challenge. In addition, over the time course of an infection, the dynamics of pathogen load and the immune response make interpretation of the contribution of different cells complex. Therefore, in this study we used a non-replicating immune challenge to the lung to study the age-related changes to the early innate response of different resident lung cells in situ.

We characterised differences in the early inflammatory response following in vivo LPS challenge of airways of young and aged mice. Next, we used precision cut lung slices (PCLS) to study the response to LPS of resident lung cells from aged and young mice within the intact architecture of the lung tissue [23, 24]. The age-related differences in responses of AMs and non-haematopoietic cells including epithelial and endothelial cells were further determined by analysing gene expression in sorted cell populations following in vivo LPS stimulation. Our work reveals that ageing results in a more pro-inflammatory phenotype in both haematopoietic and non-haematopoietic resident cells of the lung.

## Methods

### Mice

Young (2–5 months old) and aged (17–22 months old) male C57BL/6 mice (Charles River Laboratories) were maintained in specific pathogen free conditions.

### Lung challenge with LPS and tissue collection

Mice were challenged by intranasal inoculation of 100ng LPS (lipopolysaccharide from *Escherichia coli* 055: B5, InvivoGen) or Dulbecco’s phosphate buffered saline without CaCl_2_ and MgCl_2_ (Sigma-Aldrich; PBS) vehicle in a volume of 100 μl and culled four hours later. To collect bronchoalveolar lavage (BAL) the trachea was cannulated and lungs were flushed three times with 1 ml 5mM ethylenediaminetetraacetic acid (EDTA), PBS. Following centrifugation, the BAL supernatant was retained and the cells were used for flow cytometry and differential counts using cytospin preparations. Lungs were perfused with PBS *via* the right heart ventricle to flush out blood. The left lung was processed to obtain a single cell suspension by digestion for 30 min at 37^o^C with 0.25 mg/ml collagenase XI (Sigma-Aldrich) and using a GentleMACS dissociator (Miltenyi Biotec). Lung cells were processed for flow cytometry.

### Differential counts of airway cells

For cytospin preparations, BAL cells were centrifuged onto glass slides at 450 rpm for 5 min using a Cytospin 4 cytocentrifuge, then fixed and stained using the Reastain Quick-Diff kit (GENTAUR Ltd), according to manufacturer’s instructions. Differential cell counts were performed on a minimum of 300 cells per sample using an Eclipse TS100 Light microscope (Nikon).

### Flow cytometric analysis of lung and airway cells

To count total cell numbers in the airways and lungs, red blood cell lysis was performed on BAL and lung cell suspensions using ACK lysis buffer (10 mM KHCO_3_, 0.1 mM EDTA, 154 mM NH_4_Cl). Cells were stained with 7-amino-actinomycin D (7-AAD) viability staining solution (1:400, BioLegend) and counted by flow cytometry on a BD Fortessa using Countbright absolute counting beads (ThermoFisher).

For characterisation of the inflammatory response following in vivo challenge, BAL and lung cell suspensions were incubated with Fc Block (5 μg/ml, BD Biosciences), followed by antibodies and LIVE/DEAD fixable near-infrared dead cell stain kit (1:500, ThermoFisher). The following antibody conjugates were used: CD45 BV711 (30-F11, 0.5 μg/ml), CD64 PE (X54-5/7.1, 2 μg/ml), CD11b BV605 (M1/70, 1 μg/ml), CD11c PB (N418, 2 μg/ml), SiglecF APC (S17007L, 1 μg/ml), Ly6G PerCP (1A8,1 μg/ml) (BioLegend). Fluorescence minus one controls were included in each experiment. Cells were fixed in fixation buffer (BioLegend) and data were acquired on a BD Fortessa flow cytometer using FACSDiva software.

### Flow cytometry to sort lung cell populations

For cell sorting experiments, lung cells were isolated following digestion with 5mg/ml dispase II (Sigma Aldrich), and using a GentleMACS dissociator, followed by red blood cell lysis as above. Cells were incubated with Fc Block (1:100, BD Biosciences), followed by antibodies and LIVE/DEAD fixable near-infrared dead cell stain kit (1:500, ThermoFisher). The following antibody conjugates were used: Ly6G PerCP (1A8, 1 μg/ml), Epcam PE-Cy7 (G8.8, 1 μg/ml), CD45 BV711 (30-F11, 0.5 μg/ml), SiglecF APC (S17007L, 1 μg/ml), CD64 PE (X54-5/7.1, 2 μg/ml), CD31 PE-Dazzle594 (MEC13.3, 1 μg/ml) (BioLegend). Fluorescence minus one controls were included in each experiment. Cells were sorted on a BD FACS Aria III using FACSDiva software. As sorting experiments were performed over several days, we ensured that at least one young and aged mouse were sorted on each day to reduce the influence of batch effects from variation in tissue processing and sorting. Following sorting, cells were lysed immediately for RNA extraction using the RNeasy Mini kit (Qiagen).

### LPS stimulation of precision cut lung slices

PCLS were prepared from lung lobes as described [25, 26]. Briefly, lungs of naïve young and aged mice were inflated *via* the trachea with 1.5 ml or 2.5 ml, respectively, of 1.5% low gelling temperature agarose at 37^o^C, then chilled in situ. 300 μm thick PCLS were cut from lobes using a Compresstome vibrating microtome (Precisionary instruments) and transferred to 24 well plates. Following overnight incubation in 2mM L-glutamine, 100 U/ml penicillin, 100 μg/ml streptomycin, DMEM, slices were washed and stimulated with LPS in a volume of 0.5 ml. Culture supernatant was collected and four PCLS were combined at random and used for RNA extraction at 4–16 h. RNA was extracted using TRizol reagent (ThermoFisher) according to manufacturer’s instructions.

### RT-qPCR

Extracted RNA was quantified using a NanoDrop ND-100 spectrophotometer and converted to cDNA using the high-capacity RNA to cDNA kit (ThermoFisher). RT-qPCR reactions were performed using the following primer/probe sets (Thermofisher): CCL2 (*Ccl2*, Mm00441242_m1), CXCL2 (*Cxcl2*, Mm00436450_m1), Interleukin 1 beta (*Il1b*, Mm00434228_m1), TNF (*Tnf*, Mm00443258_m1), TLR4 (*Tlr4*, Mm00445273_m1), Tumor necrosis factor alpha-induced protein 3 (*Tnfaip3*, Mm00437121_m1), Interleukin-1 receptor-associated kinase 3 (*Irak3*, Mm00518541_m1), Interleukin-1 receptor-associated kinase 4 (*Irak4*, Mm00459443_m1) in TaqMan Universal MasterMix II using a 7500 Fast Systems SDS v1.4 thermocycler (Applied Biosystems). Expression was normalised to the β-actin (*Actb*) housekeeping gene (Mm00607939_s1) and presented as 2^−ΔCt^.

### ELISA

Cytokines were measured in BAL supernatants and in PCLS culture medium using Duoset ELISA kits according to manufacturer’s instructions (R&D Systems). Absorbance was read using a FLUOstar Omega plate reader and Mars Data Analysis software.

### Data analyses

Flow cytometry data was analysed using FlowJo v10.8.1 (BD Biosciences). Graphs were generated and statistical tests performed using GraphPad Prism version 9. Statistical significance of differences was determined by two-way ANOVA or unpaired t-test with Welch’s correction.

## Results

### Ageing leads to an exacerbated neutrophilic inflammatory response to LPS in the lung

**Fig. 1:**
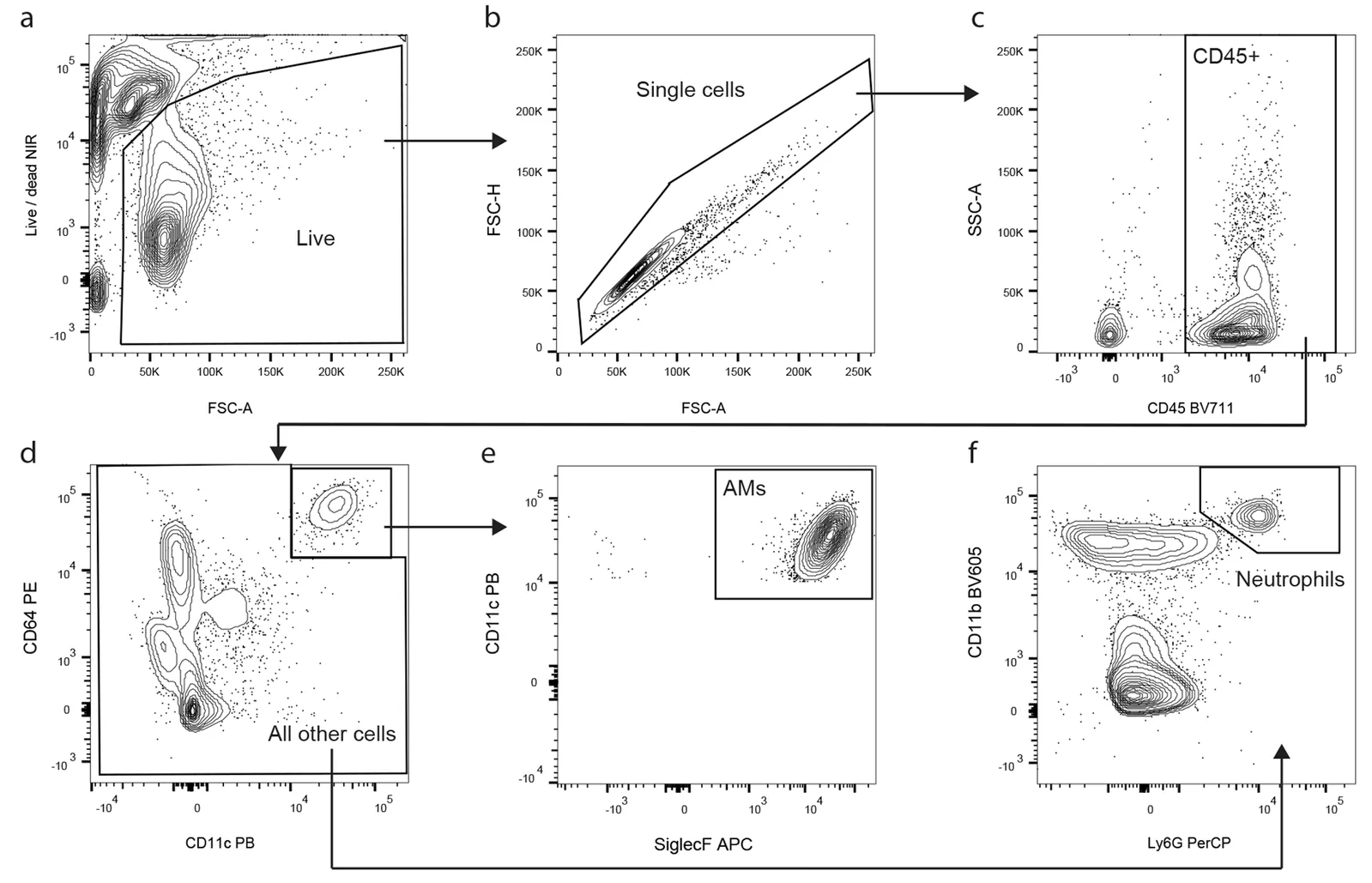
Gating strategy for the identification of inflammatory cell populations in airways and lung tissue. Lung and airway cell suspensions were stained with a viability dye (Live / dead NIR) and antibodies to surface markers followed by analysis by flow cytometry. (**a**) Live, (**b**) single, (**c**) haematopoietic (CD45+) cells were selected. Within this population, **(d**,** e**) AMs were defined as CD64 + CD11c + SiglecF + cells and (**f**) within the remaining cells, neutrophils were defined as CD11b + Ly6G + cells. The example shown is of lung cells from a young mouse following LPS challenge

To determine the intrinsic differences in the inflammatory response to innate stimuli in the lungs with age, young and aged mice were challenged intranasally with 100ng LPS in PBS or with PBS alone for four hours. BAL fluid and lung tissue were collected and single cell suspensions were analysed using flow cytometry (Fig. 1). Within the live, single, CD45 + population (Fig. 1a-c), AMs were identified as CD64 + CD11c + SiglecF + cells (Fig. 1d, e). Within the remaining cells, neutrophils were identified as CD11b + Ly6G + cells (Fig. 1f) [27]. Total cell numbers in the BAL and left lung were calculated from the live cell counts and percentage of live cells determined from the flow cytometry data analysis.

**Fig. 2:**
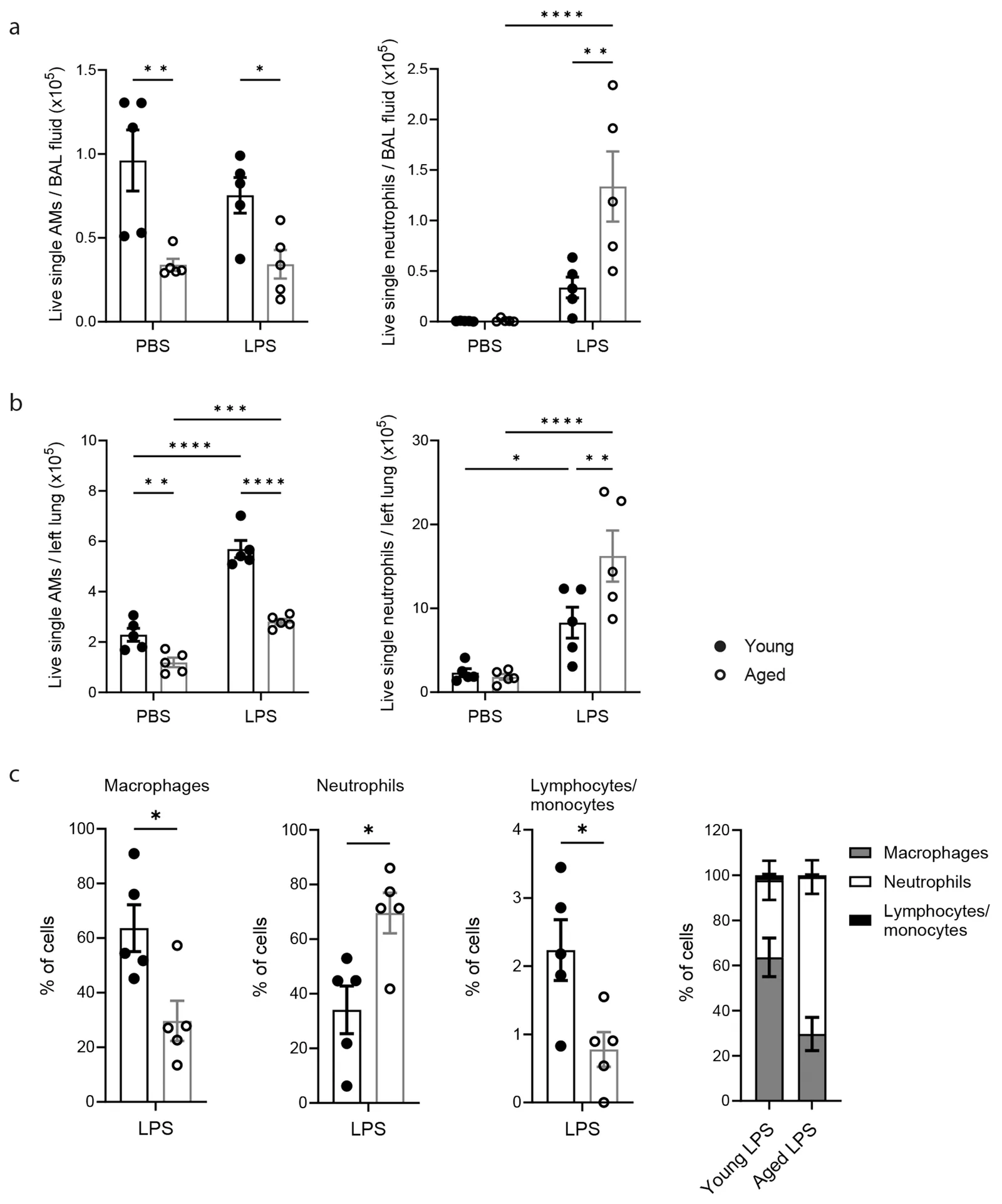
Inflammatory cell numbers in the airways and lung following LPS challenge of young and aged mice. Inflammatory cell numbers in (**a**) airway BAL fluid and (**b**) lung tissue were determined by flow cytometry 4 h after challenge with 100ng LPS or PBS vehicle in young and aged mice. Each data point represents an individual mouse and bars show mean +/- SEM (*n* = 5). Filled circles = young and open circles = aged mice. Data are representative of two independent experiments. Significance was determined by two-way ANOVA, comparing each mean with others in its age group and with others in the LPS or PBS groups. (**c**) Differential counts of airway cells obtained by bronchoalveolar lavage of young and aged mice 4 h after LPS challenge counted on cytospin preparations (*n* = 5). Significance was determined by student’s t-test. *P ≤ 0.05; **P ≤ 0.01; ***P ≤ 0.001; ****P ≤ 0.0001

In the lungs and airways, the numbers of AMs were lower both in PBS and LPS challenged aged mice than in the young (Fig. 2a, b). Following LPS challenge, this relatively low dose of LPS and early time point resulted in a recruitment of neutrophils into the airways and lung tissue in the young (Fig. 2a, b). The aged mice generated a greater inflammatory response than the young, recruiting more neutrophils into both the lung tissue and the airways.

In addition to analysis by cytometry, differential counts were observed on cytospin preparations of BAL fluid cells from LPS challenged mice (Fig. 2c). This confirmed the exacerbated neutrophilic infiltration into the airways of aged mice in which neutrophils made up a mean of 70% of airway cells four hours after LPS challenge compared to 34% in young mice.

### Ageing leads to a higher level of pro-inflammatory cytokines and chemokines in the lung in response to LPS

**Fig. 3:**
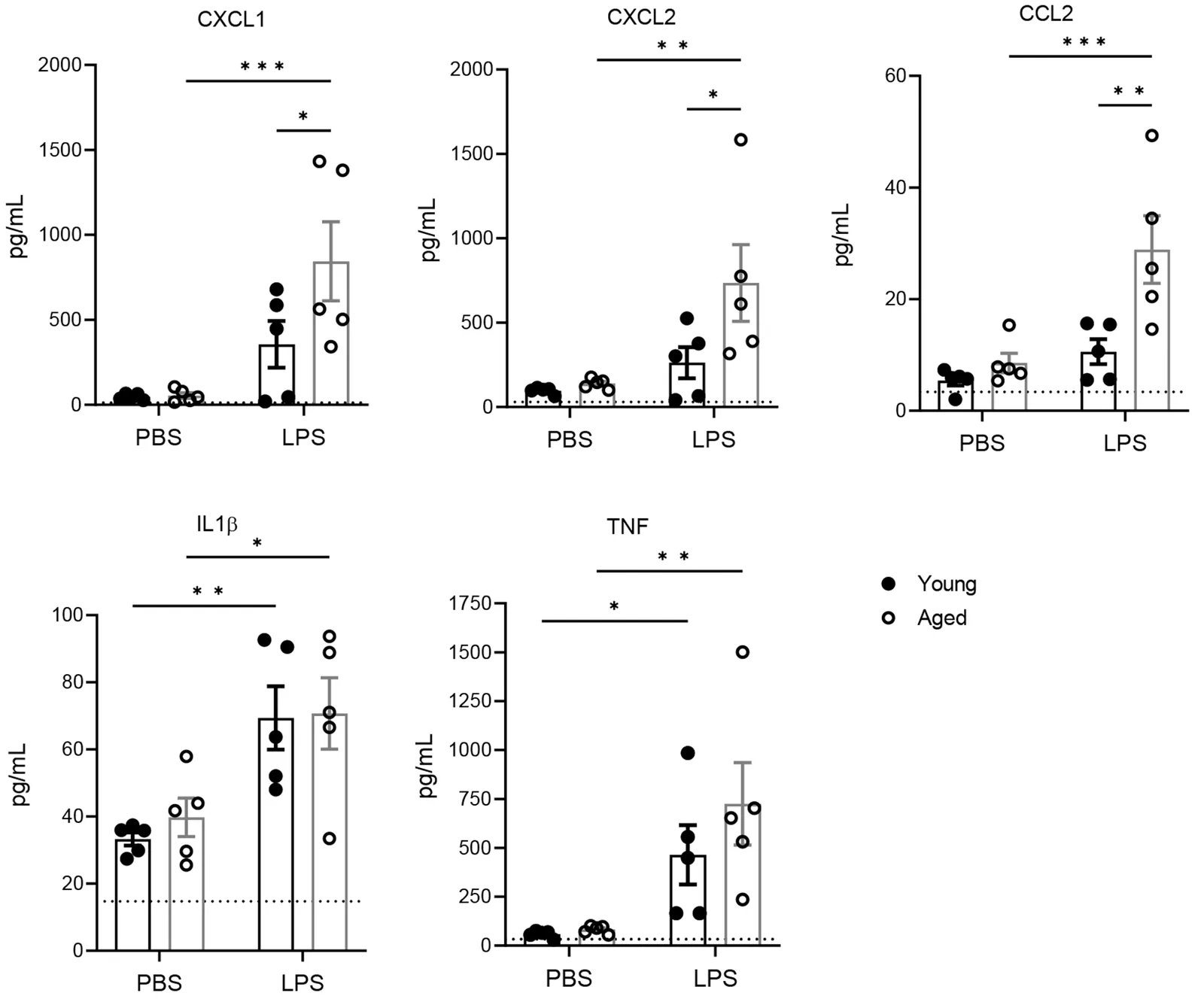
Cytokines and chemokines produced in the airways of young and aged mice following LPS challenge. Young and aged mice were challenged intranasally with 100ng LPS or PBS vehicle only. After 4 h, bronchoalveolar lavage was performed and cytokines and chemokines were measured by ELISA in BAL fluid supernatant. Each data point represents an individual mouse and bars show mean +/- SEM. Filled circles = young and open circles = aged mice. Data are representative of two independent experiments. The lower limit of detection is shown as a dotted line. Significance was determined by two-way ANOVA, comparing each mean with others in its age group and with others in the LPS or PBS groups. *P ≤ 0.05; **P ≤ 0.01; ***P ≤ 0.001; ****P ≤ 0.0001

To determine whether the higher numbers of neutrophils in the lungs of aged mice were associated with the production of inflammatory cytokines and chemokines, these were measured by ELISA in BAL fluid. In PBS challenged mice, cytokine concentrations in BAL fluid did not differ between young and aged mice. Following LPS instillation, aged mice but not young mice produced significantly higher levels of CXCL1, CXCL2 and CCL2 than in PBS challenged mice, and the amounts were significantly higher in aged than in young LPS-challenged mice (Fig. 3). Both groups significantly increased the secretion of IL-1β and TNF in response to LPS challenge and this did not significantly differ between age groups.

### Resident lung cells of aged mice show an exacerbated response to LPS

The elevated neutrophilic inflammatory response in the lungs of aged mice in response to LPS could result from intrinsic alterations in resident lung cells, in inflammatory cells recruited to the lung, and in their interactions. To determine whether resident lung cells were intrinsically different in their response to LPS in aged compared to young mice, PCLS were stimulated ex vivo.

**Fig. 4:**
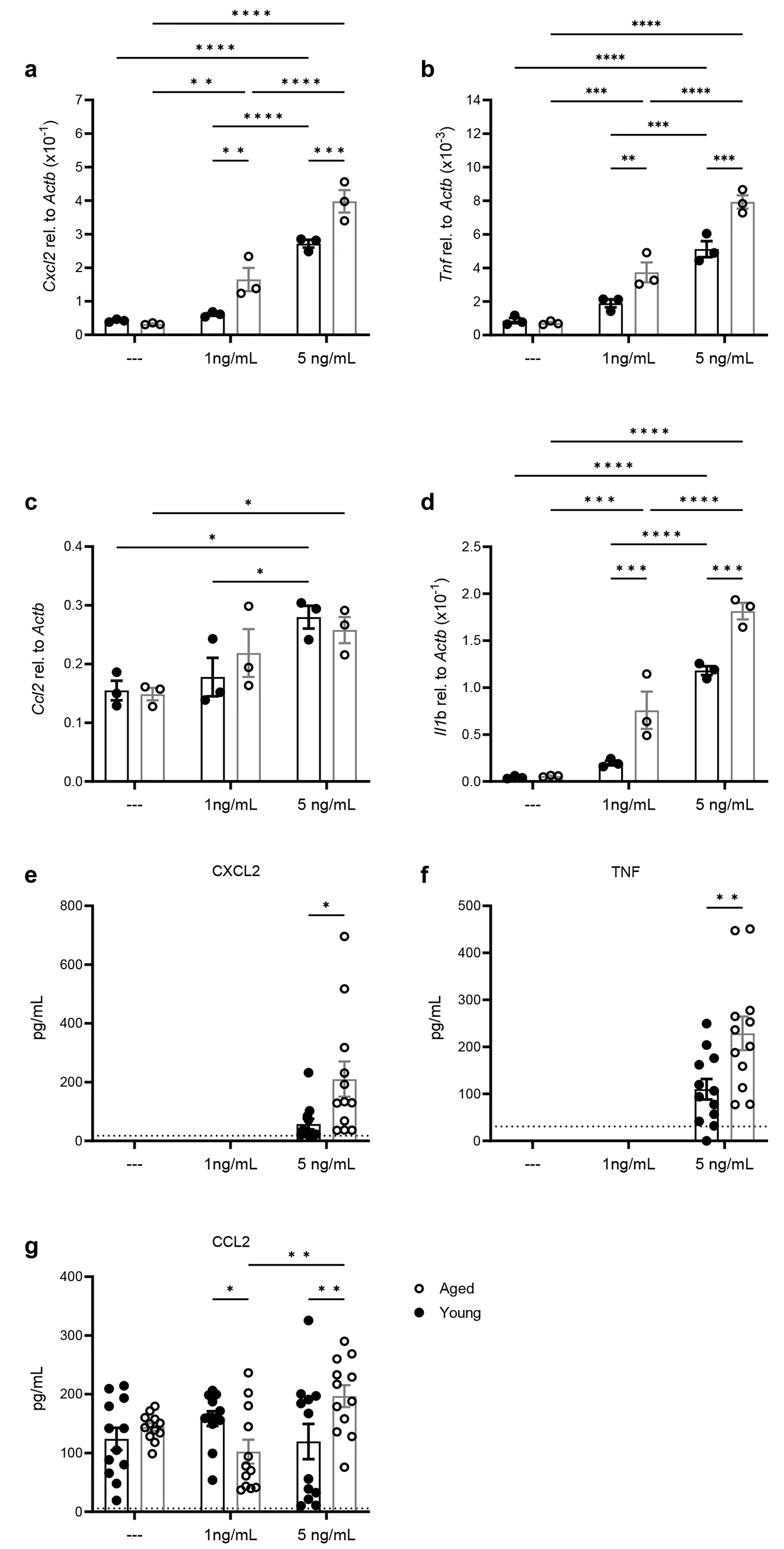
Cytokine production in precision cut lung slices from young and aged mice in response to LPS. PCLS were prepared from lung lobes of young and aged mice and stimulated with LPS for 16 h. (**a-d**) 4 PCLS within each group were randomly pooled to generate each data point and RT-PCR for cytokine gene expression was performed and normalised to *Actb*. (**e-g**) Cytokine production of stimulated PCLS was measured in culture supernatants by ELISA. Data points show individual wells. Filled circles = young and open circles = aged mice. Bars show mean +/- SEM. Data are representative of three independent experiments. Significance was determined by two-way ANOVA, comparing means within each age group and within each dose of LPS, except for **e** and **f** where a student’s t-test was performed. *P ≤ 0.05; **P ≤ 0.01; ***P ≤ 0.001; ****P ≤ 0.0001

Single PCLS generated from young and aged mice were stimulated with LPS in 24 well plates for 16 h. We found that while cytokine gene expression could be detected in PCLS at 4 h, the use of a 16 h time point was optimal for a dose response of gene expression and cytokine production to be measured across a concentration range of LPS (data not shown). Gene expression analysis of cytokines and chemokines in the lung slices at 16 h gave a dose response to LPS in both young and aged mice (Fig. 4). Expression of *Cxcl2*,* Tnf* and *Il1b* were all higher in the aged mice than in young at both doses of LPS, but not in unstimulated PCLS, and *Ccl2* was only modestly induced by LPS in lung slices from either group at this time point (Fig. 4a-d). Analysis of cytokine proteins in the culture supernatants also demonstrated a higher level of secreted CXCL2 and TNF by aged lung PCLS compared to young at the highest dose of LPS, and little change in CCL2 secretion with LPS stimulation, in agreement with the gene expression data. IL-1β protein was not detected (not shown). In summary, resident lung cells of aged mice produced significantly more neutrophil chemoattractant chemokines following LPS stimulation of PCLS than those from the lungs of young mice.

### Ageing leads to a more pro-inflammatory response in AMs and lung epithelial cells following LPS challenge in vivo

In order to determine the age-related differences in gene expression in resident innate immune cells, four hours after intranasal challenge with LPS or PBS, epithelial cells, endothelial cells, AMs and other non-haematopoeitic cells were FACS sorted from the lungs. Firstly, live, single cells were selected and the Ly6G positive population was excluded to remove recruited neutrophils (Supplementary Fig. 1). AMs were identified as CD45 + CD64 + SiglecF + cells. Within the non-haematopoeitic CD45- cell population, epithelial cells were identified as Epcam + cells, endothelial cells were identified as Epcam-CD31 + cells and other non-haematopoeitic cells were identified as Epcam-CD31- cells. Purities obtained were; 89.7 +/- 4.8% for AMs, 93.6 +/- 4.7% for epithelial cells, 93.9 +/- 4.2% for endothelial cells, and 89.9 +/- 7.1% for other non-haematopoietic cells.

RT-qPCR was performed on sorted cell populations. We first determined whether differences in the expression of the LPS receptor *Tlr4* or components of the TLR signalling pathway, *Irak3*,* Irak4* and *Tnfaip3*, could explain the differences observed in the response to LPS. Expression of these genes was not significantly different in the control, PBS challenged aged and young mice (Supplementary Fig. 2).

**Fig. 5:**
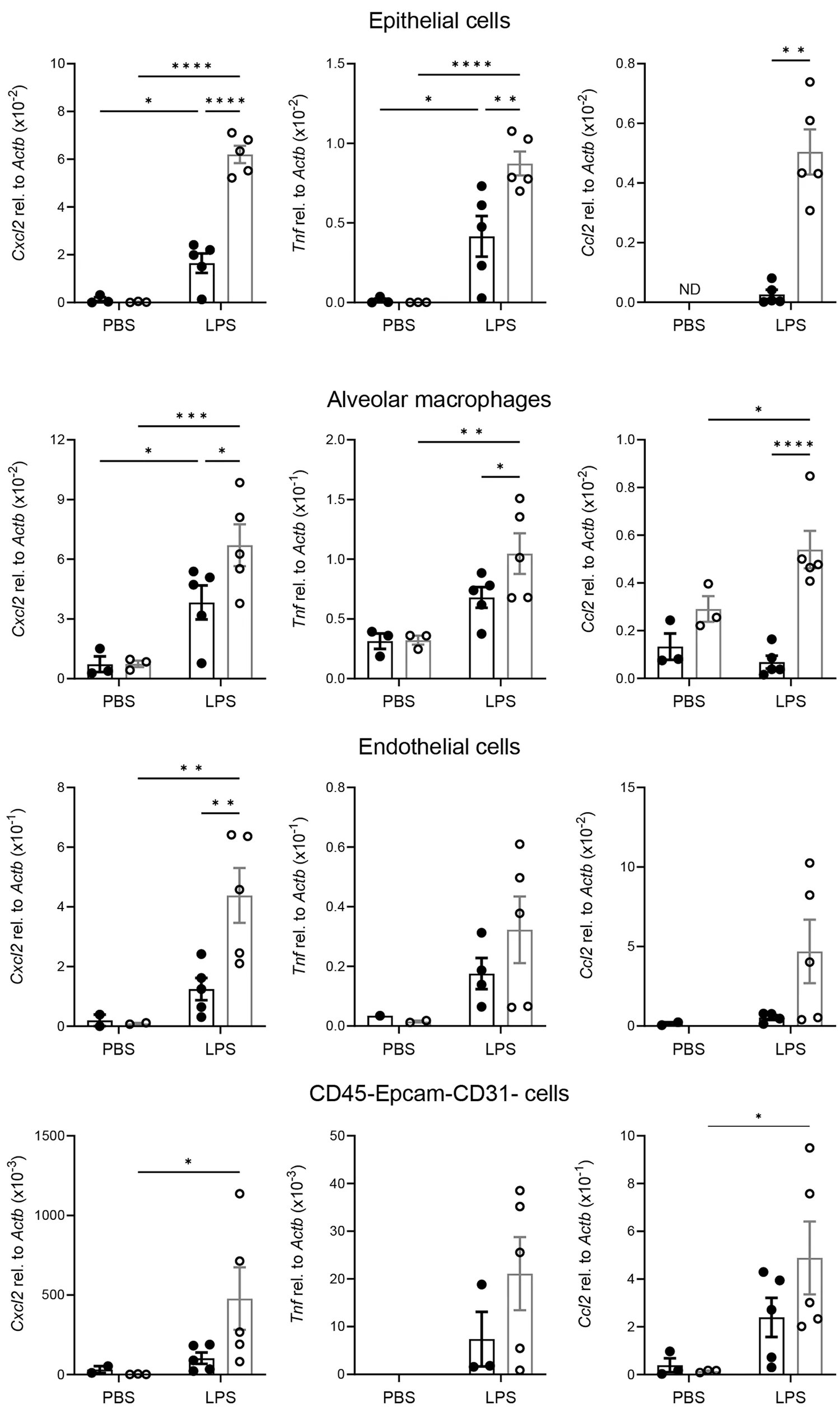
Cytokine and chemokine gene expression in resident lung cells of young and aged mice following LPS challenge. Young and aged mice were challenged intranasally with 100ng LPS (*n* = 5) or PBS only (*n* = 3) and after four hours, resident lung cell populations were FACS sorted. Gene expression was determined by RT-PCR and normalised to *Actb* expression. Data points show individual mice and are not shown where gene expression was below the limit of detection. Filled circles = young and open circles = aged mice. Bars show mean +/- SEM. Significance was determined by two-way ANOVA, comparing means within each age group and within LPS or PBS groups, except where insufficient numbers of samples reached detectable levels, and student’s t-test was performed. *P ≤ 0.05; **P ≤ 0.01; ***P ≤ 0.001; ****P ≤ 0.0001

Gene expression of *Cxcl2*,* Ccl2* and *Tnf* was compared between lungs of young and aged mice (Fig. 5). No differences in gene expression were found in the PBS challenged control groups in any of the cell populations. Following LPS challenge, expression of *Cxcl2*,* Ccl2* and *Tnf* significantly increased in epithelial cells of young and aged mice and was significantly higher in cells of LPS challenged aged mice than in those of the young. Similarly, following LPS challenge there was a significant increase in *Cxcl2*,* Ccl2* and *Tnf* expression in AMs of aged mice and expression was significantly higher in LPS challenged aged mice than in young. Following LPS challenge, endothelial cells of aged mice exhibited significantly higher *Cxcl2* expression than those of young mice and expression of *Tnf* and *Ccl2* followed the same trend but did not reach statistical significance. There was also an increased cytokine expression in other non-haematopoietic cells in LPS challenged aged mice but not in the young, but the difference between age groups did not reach statistical significance.

To compare our data with the observations of others in aged mice in the absence of LPS stimulation, we searched for our genes of interest (*Tnf*,* Il1b*,* Ccl2*,* Cxcl2*,* Cxcl1*,* Tlr4*,* Irak3*,* Irak4*,* Tnfaip3*) and other genes that could influence inflammatory responses in the lung (*Tlr3*,* Il6*, *Il18*,* Ccl3*,* Il10*) within published gene expression analysis from lung cells of aged mice. Volcano plots were used to show gene expression within in silico identified lung cell populations using scRNAseq data, and in bulk RNAseq data analysis of sorted lung macrophages and epithelial cells [6](Supplemental Fig S3). Within this data, our genes of interest were not significantly different between young and aged mice in any of the identified lung cell populations (FDR < 10%), with the exception of *Tnf* and *Ccl3* which were expressed at 1.65 and 1.57 fold higher levels in the aged mice, and *Il18* which was expressed at 0.65 fold that of the young in the aged in the scRNAseq analysis of AMs. These genes were not differentially expressed in the bulk RNAseq analysis of sorted lung macrophages.

## Discussion

Older adults are at greater risk of severe respiratory infection. This is likely to be a result of multiple factors including a decline in adaptive immunity, co-morbidities and age-related changes to the lung structure, microenvironment and innate immune response [3, 4]. The age-related changes to the innate response of resident lung cells are incompletely understood. Overall, our data demonstrate that aged mice produce a greater inflammatory response to lung LPS exposure than young mice, consisting of greater chemokine production by lung cells and enhanced neutrophil recruitment into the lung and airways. Resident cells from the lungs of aged mice, including AMs, epithelial and endothelial cells exhibited a more pro-inflammatory response following LPS challenge.

Consistent with our observation of a greater recruitment of neutrophils to the lungs in aged mice, higher neutrophil recruitment and chemokine expression has been observed in murine models of Influenza, *Acinetobacter baumannii* and *Streptococcus pneumoniae* infection [19, 28, 29]. However, age-related differences in pathogen load in these studies make interpretation of the results more complex, and none of these studies assess the early four-hour time point we study here. The use of a non-replicating stimulus, LPS, and the study of an early time point, allowed us to determine the response of resident cells of young and aged mice with an equal dose of LPS. There were higher levels of CXCL1, CXCL2 and CCL2 in the airways of aged compared to young mice in response to LPS at the time point studied. This suggests that aged mice produce these cytokines more rapidly and / or in greater quantities than young mice, however a time course study of the in vivo response would be needed to fully determine this. Although the chemokines CXCL1 and CXCL2 are important mediators of neutrophil recruitment to the lung, factors other than these chemokines could also promote neutrophil recruitment to the lungs in the aged mice.

The use of precision cut lung slices allowed us to study the dose response of resident lung cells from aged mice to LPS stimulation, without the potential contribution of recruited cells to the response. This method has the further advantages that it allows the retention of the structural organisation of lung tissue and the response of resident cells from the same sample can be determined at multiple time points and concentrations of stimulus. These studies demonstrated higher levels of expression and production of inflammatory cytokines from the resident lung cells of aged mice than those of the young. Interestingly, in response to LPS exposure, the lung slices from aged mice did not demonstrate a significantly higher production of CCL2, which was higher in the lungs of in vivo challenged mice at 4 h. This may be due the differences in the concentration of LPS used, the time point, or alternatively, recruited cells may be required for the exacerbated production of CCL2 in the aged lung. Similarly, *Il1b* and *Tnf* expression were significantly different ex vivo following LPS exposure of the PCLS, but measured levels of the proteins were not significantly different between young and aged mice in vivo. Again, this may reflect the concentration of LPS used and the time point, the regulation of the response by circulating cells or factors that are not fully represented ex vivo. Furthermore, the effects of the process of producing PCLS on cellular activity, and loss of barrier function, may also influence the nature of the response.

Individual lung resident populations were sorted by flow cytometry four hours after challenge with LPS to determine their differential responses in vivo with age. A higher expression of chemokines in AMs, epithelial and endothelial cells from aged mice was found. This demonstrates that multiple resident lung cells exhibit an enhanced inflammatory phenotype in vivo in aged mice. The differences in responses of resident cells could not be accounted for by increased *Tlr4* or TLR4 signalling component expression in lung cells prior to stimulation with LPS, however, the levels of these proteins would need to be directly measured to rule this possibility out. This differs from reports of higher levels of A20 (*Tnfaip3*) in the aged lung [30].

We found no age-related differences in AM or epithelial cell cytokine expression at baseline. We further compared our results to published gene expression analyses in the unchallenged, aged lung and specifically assessed the expression of our genes of interest and extended this to other genes that could influence inflammatory responses in the lung, including TLR3, the pro-inflammatory cytokines IL-6, IL-18 and CCL3, and the regulatory cytokine Il-10 (*Tnf*,* Il1b*,* Ccl2*,* Cxcl2*,* Cxcl1*,* Tlr3*,* Tlr4*,* Irak3*,* Irak4*,* Tnfaip3*,* Il6*,* Il18*,* Ccl3*,* Il10*). In agreement with our findings, analysis by Angelidis *et al.* showed no significant differences in expression of these genes in resident cell populations in the lung of aged compared to young mice, with the exception of *Tnf* and *Ccl3* expression which were modestly elevated in in silico identified aged AMs, analysed using scRNAseq, but not in FACS sorted macrophages analysed by bulk RNAseq (6). Of our genes of interest, others report higher *Tnf*, *Il6*,* Cxcl1*, *Cxcl2*,* Ccl3* and *Il1b*, and lower *Tlr4* and *Il18* expression in bulk RNAseq analysis of FACS sorted aged AMs [8], higher *Ccl2*, *Cxcl2*, *Il1b*, *Cxcl1* and *Ccl3* in microarray analysis of FACS purified aged AMs [9] and higher *Ccl2*, *Tnf* and *Il10* expression in PCR analysis of aged AMs isolated by lavage compared to AMs from young mice [14]. Differences between studies may derive from the age and housing conditions of the mice, cell isolation processes and gene expression methodology.

We find reduced numbers of AMs in the lungs with age which is consistent with reports of reduced self-renewal capacity in these cells with age [8, 9]. Ex vivo studies of responses of AMs from aged mice have been contradictory, with some reporting enhanced cytokine responses in these cells and others finding a reduced response with age [11–13]. To the best of our knowledge, this is the first study to investigate age-associated functional alterations in AMs following in vivo LPS challenge, and we find enhanced inflammatory cytokine production from these cells.

We report a greater inflammatory cytokine gene expression in lung epithelial cells 4 h following LPS challenge. Others have reported higher expression of cytokines and chemokines in AEC2 from aged mice challenged with LPS at 24 h, and 6 days after influenza virus infection [18, 19]. However, a lower response to house dust mite extract in tracheal epithelial cells from older mice stimulated ex vivo was also reported [31]. Age-related differences in the inflammatory response in epithelial cells may be influenced by the time point, the nature of the stimulant and the source and composition of epithelial cells studied [3]. We sorted all lung epithelial cells for this analysis, and there is potential heterogeneity within that population that may be altered with age [6]. Furthermore, during ageing, senescent cells accumulate in tissues. These arrested cells are characterised by secretion of the senescence associated secretory phenotype, which includes production of pro-inflammatory cytokines. They may contribute to the low-level chronic inflammation seen with age known as ‘inflammaging’ that may drive age-associated conditions, including those of the lung [3, 32–35]. The presence of a higher frequency of senescent epithelial cells in aged mice than in young [19, 36] may contribute to inflammation, either directly or indirectly by influencing the responses or phenotype of other resident cells and this warrants further study.

We also observe age-related differences in the response of lung endothelial cells in the aged mice. To our knowledge, this is the first study of how the cytokine and chemokine responses of lung endothelial cells are influenced by age and we find that they are more pro-inflammatory than the young. An impaired ability of aged lung endothelial cells to regenerate and promote resolution of inflammation in the lung was reported in mouse sepsis models [22]. As lung endothelial cells can regulate inflammatory responses in the lung during infection, alteration in their immune function with age is an area that merits further study [20, 21].

Although we have used LPS as a stimulant to mimic acute respiratory infection in our model, its receptor TLR4 and its ligands have been implicated in the chronic inflammation associated with ageing and with age-related diseases in other tissues [37, 38]. For example, signals from the microbiota may promote inflammation that increases myeloid cell differentiation of haematopoietic stem cells, which in turn promotes immunosenescence [39, 40]. TLR4 has also been implicated in age-related neurodegenerative diseases [38], adipose inflammation and poor glucose tolerance, and cardiovascular disease [41, 42]. Therefore, the changes to TLR4 mediated responses with age may have wider implications for lung ageing and age-related disease. Limitations of our study include the use of only a single sex, male, and single strain of mice, C57BL/6. Therefore, further work will be needed to determine the wider relevance of these results to aging and age-related diseases.

## Conclusions

In summary, we have demonstrated that ageing leads to an enhanced neutrophilic inflammation in the lung following LPS exposure, with elevated production of pro-inflammatory cytokines and chemokines from haematopoietic and non-haematopoietic resident lung cells. Our work demonstrated that multiple resident cells can contribute to exacerbated lung pathology and inflammation *via* early chemokine production following infectious challenge in the lung. Further understanding of the cellular mechanisms that lead to these age-related differences, such as metabolic changes to the cells, warrant further study. The age-associated changes to innate immunity we describe have important implications for our understanding of the pathogenesis of respiratory infections in the elderly and for the development of novel therapies for the burden of age-related respiratory disease [43, 44].

## Electronic supplementary material

Below is the link to the electronic supplementary material.

Supplementary Material 1

## Data Availability

The datasets used and/or analysed during the current study are available from the corresponding author on reasonable request.

## Abbreviations

AEC2: Type II alveolar epithelial cells
AM: Alveolar macrophages
BAL: Bronchoalveolar lavage
LPS: Lipopolysaccharide (LPS)
PCLS: Precision cut lung slices
TNF: Tumor necrosis factor

## References

[1] Häder A, Köse-Vogel N, Schulz L, Mlynska L, Hornung F, Hagel S, et al. Respiratory infections in the aging lung: implications for diagnosis, therapy, and prevention. Aging Dis. 2023;14(4):1091–104. 10.14336/AD.2023.0329 PMC10389836 37163442

[2] Prevalence and attributable health burden of chronic respiratory diseases. 1990–2017: a systematic analysis for the global burden of disease study 2017. Lancet Respir Med. 2020;8(6):585–96. 10.1016/S2213-2600(20)30105-3 PMC7284317 32526187

[3] Schneider JL, Rowe JH, Garcia-de-Alba C, Kim CF, Sharpe AH, Haigis MC. The aging lung: physiology, disease, and immunity. Cell. 2021;184(8):1990–2019. 10.1016/j.cell.2021.03.005 PMC8052295 33811810

[4] Cho SJ, Stout-Delgado HW. Aging and lung disease. Annu Rev Physiol. 2020;82:433–59. 10.1146/annurev-physiol-021119-034610 PMC7998901 31730381

[5] Krausgruber T, Fortelny N, Fife-Gernedl V, Senekowitsch M, Schuster LC, Lercher A, et al. Structural cells are key regulators of organ-specific immune responses. Nature. 2020;583(7815):296–302. 10.1038/s41586-020-2424-4 PMC7610345 32612232

[6] Angelidis I, Simon LM, Fernandez IE, Strunz M, Mayr CH, Greiffo FR, et al. An atlas of the aging lung mapped by single cell transcriptomics and deep tissue proteomics. Nat Commun. 2019;10(1):963. 10.1038/s41467-019-08831-9 PMC6393476 30814501

[7] Bissonnette EY, Lauzon-Joset JF, Debley JS, Ziegler SF. Cross-talk between alveolar macrophages and lung epithelial cells is essential to maintain lung homeostasis. Front Immunol. 2020;11:583042. 10.3389/fimmu.2020.583042 PMC7593577 33178214

[8] McQuattie-Pimentel AC, Ren Z, Joshi N, Watanabe S, Stoeger T, Chi M et al. The lung microenvironment shapes a dysfunctional response of alveolar macrophages in aging. J Clin Investig. 2021;131(4). 10.1172/JCI140299 PMC7919859 33586677

[9] Wong CK, Smith CA, Sakamoto K, Kaminski N, Koff JL, Goldstein DR. Aging impairs alveolar macrophage phagocytosis and increases influenza-induced mortality in mice. J Immunol. 2017;199(3):1060–8. 10.4049/jimmunol.1700397 PMC5557035 28646038

[10] Li Z, Jiao Y, Fan EK, Scott MJ, Li Y, Li S, et al. Aging-impaired filamentous actin polymerization signaling reduces alveolar macrophage phagocytosis of bacteria. J Immunol. 2017;199(9):3176–86. 10.4049/jimmunol.1700140 PMC5679440 28947541

[11] Boyd AR, Shivshankar P, Jiang S, Berton MT, Orihuela CJ. Age-related defects in TLR2 signaling diminish the cytokine response by alveolar macrophages during murine pneumococcal pneumonia. Exp Gerontol. 2012;47(7):507–18. 10.1016/j.exger.2012.04.004 PMC3368096 22548913

[12] Higashimoto Y, Fukuchi Y, Shimada Y, Ishida K, Ohata M, Furuse T, et al. The effects of aging on the function of alveolar macrophages in mice. Mech Ageing Dev. 1993;69(3):207–17. 10.1016/0047-6374(93)90024-l 8412370

[13] Kohut ML, Senchina DS, Madden KS, Martin AE, Felten DL, Moynihan JA. Age effects on macrophage function vary by tissue site, nature of stimulant, and exercise behavior. Exp Gerontol. 2004;39(9):1347–60. 10.1016/j.exger.2004.07.001 15489058

[14] Lafuse WP, Rajaram MVS, Wu Q, Moliva JI, Torrelles JB, Turner J, et al. Identification of an increased alveolar macrophage subpopulation in old mice that displays unique inflammatory characteristics and is permissive to Mycobacterium tuberculosis infection. J Immunol. 2019;203(8):2252–64. 10.4049/jimmunol.1900495 PMC6783358 31511357

[15] Hewitt RJ, Lloyd CM. Regulation of immune responses by the airway epithelial cell landscape. Nat Rev Immunol. 2021;21(6):347–62. 10.1038/s41577-020-00477-9 PMC7804588 33442032

[16] Watson JK, Sanders P, Dunmore R, Rosignoli G, Julé Y, Rawlins EL, et al. Distal lung epithelial progenitor cell function declines with age. Sci Rep. 2020;10(1):10490. 10.1038/s41598-020-66966-y PMC7319976 32591591

[17] Ortega-Martínez M, Rodríguez-Flores LE, Ancer-Arellano A, Cerda-Flores RM, de-la-Garza-González C, Ancer-Rodríguez J, et al. Analysis of cell turnover in the bronchiolar epithelium through the normal aging process. Lung. 2016;194(4):581–7. 10.1007/s00408-016-9890-3 27164984

[18] Yazicioglu T, Mühlfeld C, Autilio C, Huang C-K, Bär C, Dittrich-Breiholz O, et al. Aging impairs alveolar epithelial type II cell function in acute lung injury. Am J Physiol Lung Cell Mol Physiol. 2020;319(5):L755–69. 10.1152/ajplung.00093.2020 32877222

[19] Kulkarni U, Zemans RL, Smith CA, Wood SC, Deng JC, Goldstein DR. Excessive neutrophil levels in the lung underlie the age-associated increase in influenza mortality. Mucosal Immunol. 2019;12(2):545–54. 10.1038/s41385-018-0115-3 PMC6375784 30617300

[20] Teijaro John R, Walsh Kevin B, Cahalan S, Fremgen Daniel M, Roberts E, Scott F, et al. Endothelial cells are central orchestrators of cytokine amplification during influenza virus infection. Cell. 2011;146(6):980–91. 10.1016/j.cell.2011.08.015 PMC3176439 21925319

[21] Major J, Crotta S, Finsterbusch K, Chakravarty P, Shah K, Frederico B, et al. Endothelial AHR activity prevents lung barrier disruption in viral infection. Nature. 2023;621(7980):813–20. 10.1038/s41586-023-06287-y PMC7615136 37587341

[22] Huang X, Zhang X, Machireddy N, Evans CE, Trewartha SD, Hu G, et al. Endothelial FoxM1 reactivates aging-impaired endothelial regeneration for vascular repair and resolution of inflammatory lung injury. Sci Transl Med. 2023;15(709):eabm5755. 10.1126/scitranslmed.abm5755 PMC10894510 37585502

[23] Liu G, Betts C, Cunoosamy DM, Åberg PM, Hornberg JJ, Sivars KB, et al. Use of precision cut lung slices as a translational model for the study of lung biology. Respir Res. 2019;20(1):162. 10.1186/s12931-019-1131-x PMC6642541 31324219

[24] Viana F, O’Kane CM, Schroeder GN. Precision-cut lung slices: a powerful ex vivo model to investigate respiratory infectious diseases. Mol Microbiol. 2022;117(3):578–88. 10.1111/mmi.14817 PMC9298270 34570407

[25] Michalaki C, Dean C, Johansson C. The use of precision-cut lung slices for studying innate immunity to viral infections. Curr Protocols. 2022;2(8):e505. 10.1002/cpz1.505 PMC9545600 35938685

[26] Kim SY, Mongey R, Griffiths M, Hind M, Dean CH. An ex vivo acid injury and repair (AIR) model using precision-cut lung slices to understand lung injury and repair. Curr Protocols Mouse Biology. 2020;10(4):e85. 10.1002/cpmo.85 33217226

[27] Misharin AV, Morales-Nebreda L, Mutlu GM, Budinger GRS, Perlman H. Flow cytometric analysis of macrophages and dendritic cell subsets in the mouse lung. Am J Respir Cell Mol Biol. 2013;49(4):503–10. 10.1165/rcmb.2013-0086MA PMC3824047 23672262

[28] Bou Ghanem EN, Clark S, Du X, Wu D, Camilli A, Leong JM, et al. The α-tocopherol form of vitamin E reverses age-associated susceptibility to streptococcus pneumoniae lung infection by modulating pulmonary neutrophil recruitment. J Immunol. 2015;194(3):1090–9. 10.4049/jimmunol.1402401 PMC4834212 25512603

[29] Gu H, Liu D, Zeng X, Peng LS, Yuan Y, Chen ZF, et al. Aging exacerbates mortality of Acinetobacter baumannii pneumonia and reduces the efficacies of antibiotics and vaccine. Aging. 2018;10(7):1597–608. 10.18632/aging.101495 PMC6075437 30018181

[30] Hinojosa CA, Akula Suresh Babu R, Rahman MM, Fernandes G, Boyd AR, Orihuela CJ. Elevated A20 contributes to age-dependent macrophage dysfunction in the lungs. Exp Gerontol. 2014;54:58–66. 10.1016/j.exger.2014.01.007 PMC3989429 24440463

[31] Schiffers C, Lundblad LKA, Hristova M, Habibovic A, Dustin CM, Daphtary N, et al. Downregulation of DUOX1 function contributes to aging-related impairment of innate airway injury responses and accelerated senile emphysema. Am J Physiol Lung Cell Mol Physiol. 2021;321(1):L144–58. 10.1152/ajplung.00021.2021 PMC8321859 33951398

[32] Hansel C, Jendrossek V, Klein D. Cellular senescence in the lung: the central role of senescent epithelial cells. Int J Mol Sci. 2020;21(9). 10.3390/ijms21093279 PMC7247355 32384619

[33] Liu Z, Liang Q, Ren Y, Guo C, Ge X, Wang L, et al. Immunosenescence: molecular mechanisms and diseases. Signal Transduct Target Therapy. 2023;8(1):200. 10.1038/s41392-023-01451-2 PMC10182360 37179335

[34] Torrance BL, Haynes L. Cellular senescence is a key mediator of lung aging and susceptibility to infection. Front Immunol. 2022;13:1006710. 10.3389/fimmu.2022.1006710 PMC9473698 36119079

[35] Ferrucci L, Fabbri E. Inflammageing: chronic inflammation in ageing, cardiovascular disease, and frailty. Nat Reviews Cardiol. 2018;15(9):505–22. 10.1038/s41569-018-0064-2 PMC6146930 30065258

[36] Lehmann M, Hu Q, Hu Y, Hafner K, Costa R, van den Berg A, et al. Chronic WNT /\beta-catenin signaling induces cellular senescence in lung epithelial cells. Cell Signal. 2020;70:109588. 10.1016/j.cellsig.2020.109588 PMC8968687 32109549

[37] Kim H-J, Kim H, Lee J-H, Hwangbo C. Toll-like receptor 4 (TLR4): new insight immune and aging. Immun Ageing. 2023;20(1):67. 10.1186/s12979-023-00383-3 PMC10668412 38001481

[38] Tang D, Kang R, Zeh HJ, Lotze MT. The multifunctional protein HMGB1: 50 years of discovery. Nat Rev Immunol. 2023;23(12):824–41. 10.1038/s41577-023-00894-6 37322174

[39] Kovtonyuk LV, Caiado F, Garcia-Martin S, Manz EM, Helbling P, Takizawa H, et al. IL-1 mediates microbiome-induced inflammaging of hematopoietic stem cells in mice. Blood. 2022;139(1):44–58. 10.1182/blood.2021011570 34525198

[40] Ross JB, Myers LM, Noh JJ, Collins MM, Carmody AB, Messer RJ, et al. Depleting myeloid-biased haematopoietic stem cells rejuvenates aged immunity. Nature. 2024;628(8006):162–70. 10.1038/s41586-024-07238-x PMC11870232 38538791

[41] Ghosh AK, O’Brien M, Mau T, Yung R. Toll-like receptor 4 (TLR4) deficient mice are protected from adipose tissue inflammation in aging. Aging. 2017;9(9):1971–82. 10.18632/aging.101288 PMC5636669 28898202

[42] Liu H, Chu S, Wu Z. Loss of toll-like receptor 4 ameliorates cardiovascular dysfunction in aged mice. Immun Ageing. 2021;18(1):42. 10.1186/s12979-021-00251-y PMC8569991 34740366

[43] Campisi J, Kapahi P, Lithgow GJ, Melov S, Newman JC, Verdin E. From discoveries in ageing research to therapeutics for healthy ageing. Nature. 2019;571(7764):183–92. 10.1038/s41586-019-1365-2 PMC7205183 31292558

[44] Li X, Li C, Zhang W, Wang Y, Qian P, Huang H. Inflammation and aging: signaling pathways and intervention therapies. Signal Transduct Target Therapy. 2023;8(1):239. 10.1038/s41392-023-01502-8 PMC10248351 37291105

