# Correction: Ageing results in an exacerbated inflammatory response to LPS by resident lung cells

**DOI:** 10.1186/s12979-026-00579-3

**Published:** 2026-10-03

**Authors:** Celia Diaz-Nicieza, Laura Sahyoun, Christina Michalaki, Cecilia Johansson, Fiona J. Culley

**Affiliations:** National Heart and Lung Institute, Faculty of Medicine, Imperial College London, Norfolk Place, London, W2 1PG UK

**Correction: Immun Ageing 21, 62 (2024)**

**https://doi.org/10.1186/s12979-024-00467-8**

Following the publication of the original article [1], the sentence “For cell sorting experiments, lung cells were isolated as above, incubated with Fc Block (1:100, BD Biosciences), followed by antibodies and LIVE/DEAD fixable near-infrared dead cell stain kit (1:500, ThermoFisher).” in the “Flow cytometry to sort lung cell populations” section under Methods has been corrected to “For cell sorting experiments, lung cells were isolated following digestion with 5 mg/ml dispase II (Sigma Aldrich), and using a GentleMACS dissociator, followed by red blood cell lysis as above. Cells were incubated with Fc Block (1:100, BD Biosciences), followed by antibodies and LIVE/DEAD fixable near-infrared dead cell stain kit (1:500, ThermoFisher).”

The original article [1] has been updated.
