# Supplementary material for: Ageing results in an exacerbated inflammatory response to LPS by resident lung cells
Source: Immun Ageing. 2024 Sep 12;21:62. doi: 10.1186/s12979-024-00467-8 (PMC11391591; doi:10.1186/s12979-024-00467-8)
Supplement: Supplementary file 1 — Supplementary Material 1 [file 12979_2024_467_MOESM1_ESM.docx]

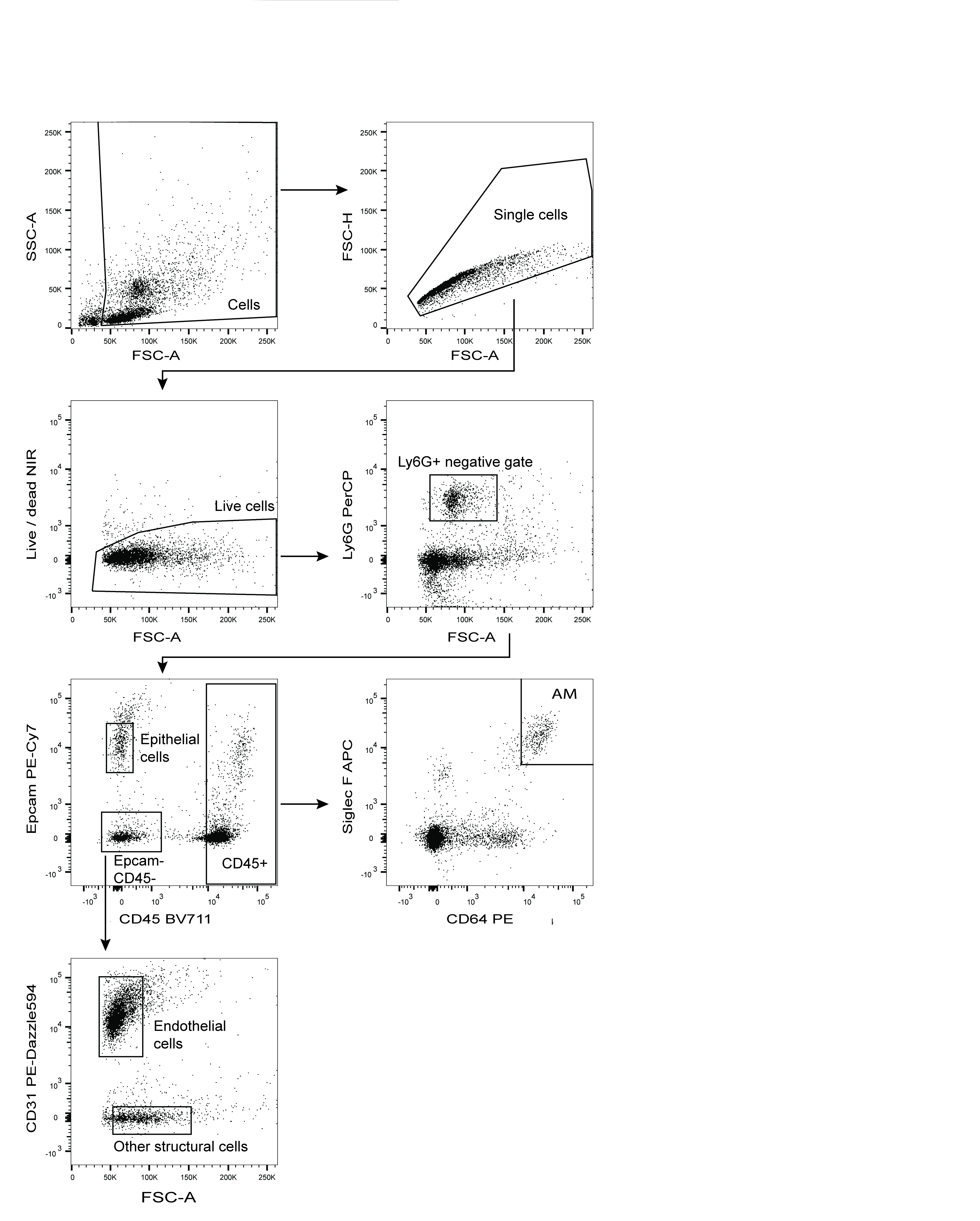


**Supplementary Figure 1. Gating strategy for FACS sorting of airway structural cells**

Mice were challenged intranasally with LPS or PBS. After four hours, lung cell suspensions were prepared by dispase digestion, stained with viability dye (Live/dead NIR) and antibodies to surface markers, and sorted by flow cytometry. Live single cells were selected and granulocytes excluded using a negative gate for Ly6G+ cells. Within the remaining cells, populations were sorted as follows; AM as CD45+CD64+SiglecF+ cells; epithelial cells as CD45- Epcam+ cells; endothelial cells as CD45-Epcam-CD31+ cells and remaining non-haematopoeitic CD45-, Epcam-, CD31- cells were retained. Data shows a representative young mouse challenged with LPS. 5000 cells are shown per plot.


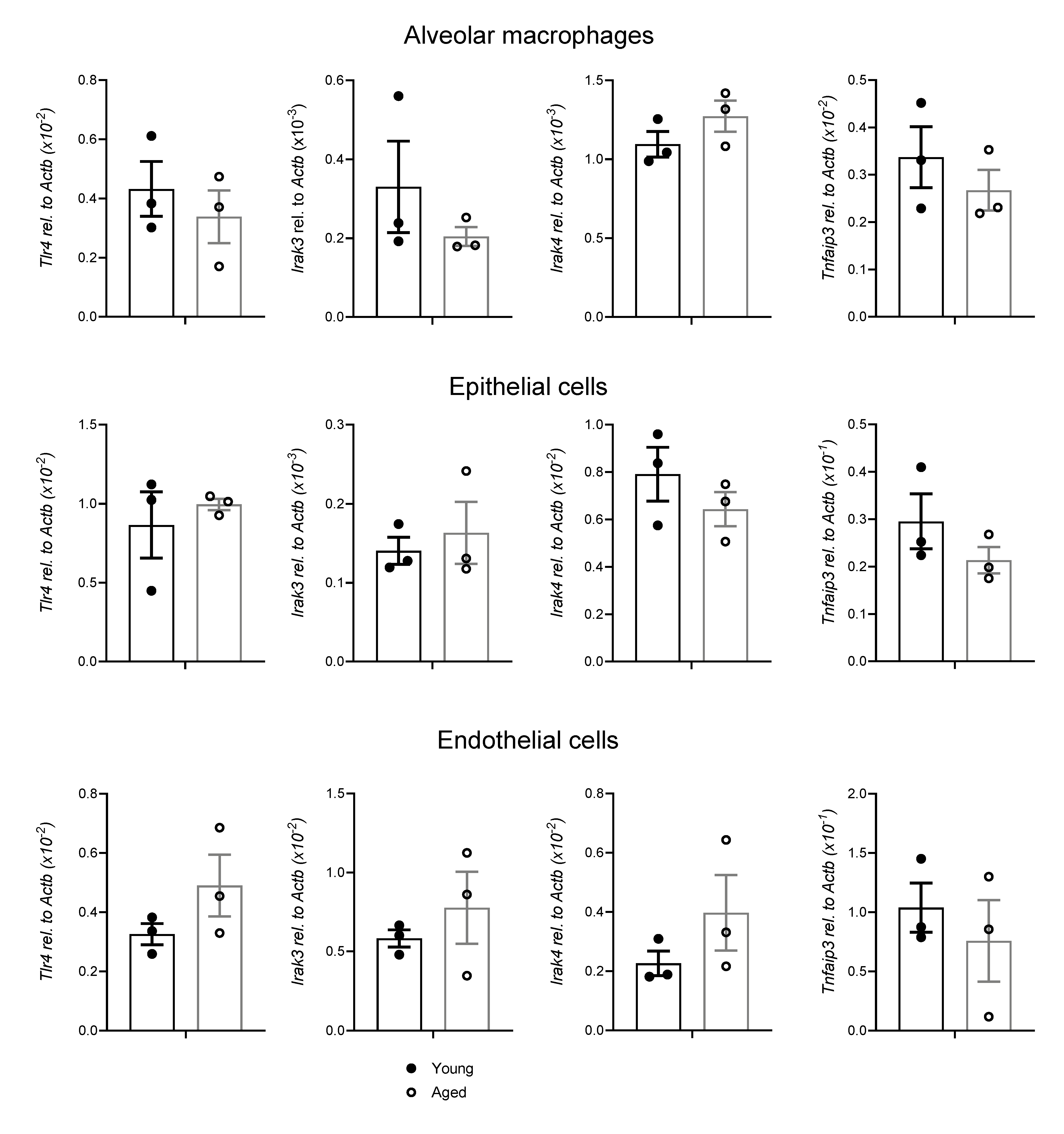


**Supplementary Figure 2. Expression of components of the TLR4 signalling pathway in resident lung cells does not differ between young and aged mice.** Epithelial cells, AM and endothelial cells from young and aged mice challenged with PBS only were FACS sorted. Expression of TLR4 signalling pathway components was determined by RT-PCR and normalised to *Actb.* Significance was tested using a student’s t-test.


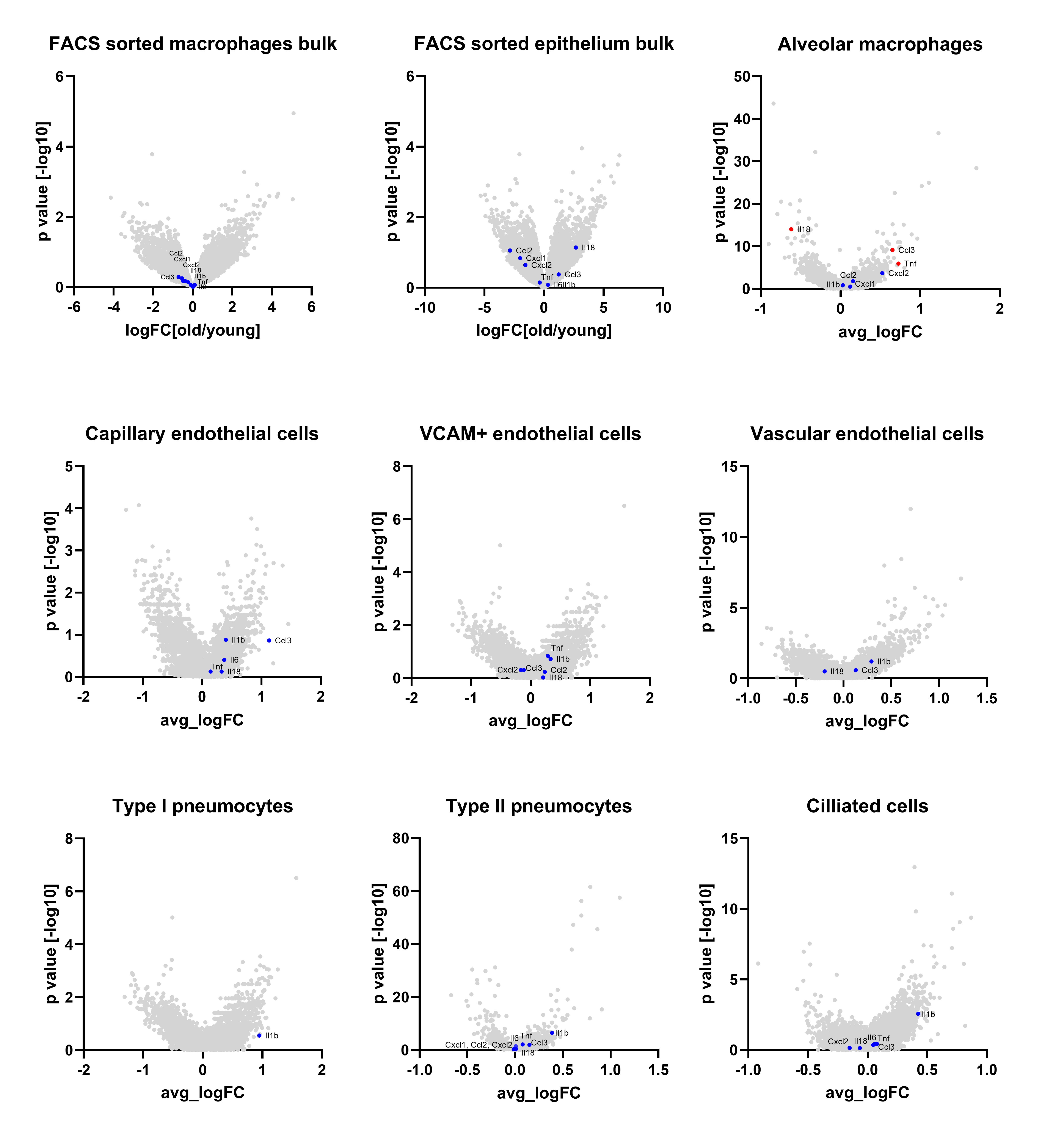


**Supplementary Figure 3**

Expression of genes of interest within published bulk RNAseq analysis of sorted lung macrophage and epithelial cells and within scRNAseq analysis of lung cells from unchallenged young and aged mice, using expression data for different *in silico* identified cell populations (1). For illustrative purposes and comparison with data presented in this manuscript, plots for alveolar macrophages, epithelial and endothelial cells are shown and expression of *Ccl2, Cxcl2, Il6, Il1b, Tnf, Il18, Cxcl1 and Ccl3* added to volcano plots as blue dots, or coloured red where the False discovery rate <0.1. All other genes are shown in light grey.

(1) Angelidis I, Simon LM, Fernandez IE, Strunz M, Mayr CH, Greiffo FR, et al. An atlas of the aging lung mapped by single cell transcriptomics and deep tissue proteomics. Nature Communications. 2019;10(1):963.
